# Hand hygiene in surgery in Benin: opportunities and challenges

**DOI:** 10.1186/s13756-020-00748-z

**Published:** 2020-06-15

**Authors:** Carine Laurence Yehouenou, Angèle Modupe Dohou, Ariane Dessièdé Fiogbe, Marius Esse, Cyriaque Degbey, Anne Simon, Olivia Dalleur

**Affiliations:** 1 Clinical Pharmacy Research Group (CLIP), Louvain Drug Research Institute (LDRI), Université catholique de Louvain UCLouvain, Brussels, Belgium; 2 Laboratoire de Référence des Mycobactéries (LRM), Cotonou, Benin; 3 Faculte des Sciences de la Sante (FSS), Université d’Abomey Calavi (UAC), Cotonou, Benin O3BP1326; 4 Institut Régional de Santé Publique Comlan Alfred Quenum (IRSP), Ouidah, Benin; 5 Clinique Universitaire d’Hygiène Hospitalière, Centre National Hospitalo-universitaire Hubert Koutoukou Maga, Cotonou, Benin; 6 Pole de microbiologie, Institut de Recherche Expérimentale et Clinique (IREC), Université catholique de Louvain UCLouvain, Brussels, Belgium; 7 Microbiologie, Cliniques universitaires Saint-Luc, Université catholique de Louvain, UCLouvain, Brussels, Belgium; 8 Pharmacy, Cliniques universitaires Saint-Luc, Université catholique de Louvain, UCLouvain, Brussels, Belgium

**Keywords:** Hand hygiene, Alcohol based hand rub, Hand washing, Surgery, Observation

## Abstract

**Background:**

Hand Hygiene (HH) has been described as the cornerstone and starting point in all infection control. Compliance to HH is a fundamental quality indicator. The aim of this study was to investigate the HH compliance among Health-care Workers (HCWs) in Benin surgical care units.

**Methods:**

A multicenter prospective observational study was conducted for two months. The World Health Organization (WHO) Hand Hygiene Observation Tool was used in obstetric and gastrointestinal surgery through six public hospitals in Benin. HH compliance was calculated by dividing the number of times HH was performed by the total number of opportunities. HH technique and duration were also observed.

**Results:**

A total of 1315 HH opportunities were identified during observation period. Overall, the compliance rate was 33.3% (438/1315), without significant difference between professional categories (nurses =34.2%; auxiliaries =32.7%; and physicians =32.4%; *p* = 0.705). However, compliance rates differed (*p* < 0.001) between obstetric (49.4%) and gastrointestinal surgery (24.3%). Generally, HCWs were more compliant after body fluid exposure (54.5%) and after touching patient (37.5%), but less before patient contact (25.9%) and after touching patient surroundings (29.1%).

HCWs were more likely to use soap and water (72.1%) compared to the alcohol based hand rub solution (27.9%). For all of the WHO five moments, hand washing was the most preferred action. For instance, hand rub only was observed 3.9% after body fluid exposure and 16.3% before aseptic action compared to hand washing at 50.6 and 16.7% respectively. Duration of HH performance was not correctly adhered to 94% of alcohol hand rub cases (mean duration 9 ± 6 s instead of 20 to 30 s) and 99.5% of hand washing cases (10 ± 7 s instead of the recommended 40 to 60 s). Of the 432 HCWs observed, 77.3% followed HH prerequisites (i.e. no artificial fingernails, no jewellery). We also noted a lack of permanent hand hygiene infrastructures such as sink, soap, towels and clean water.

**Conclusion:**

Compliance in surgery was found to be low in Benin hospitals. They missed two opportunities out of three to apply HH and when HH was applied, technique and duration were not appropriate. HH practices should be a priority to improve patient safety in Benin.

## Background

Healthcare-Associated Infections (HAIs) are a major problem for patient safety. They are related to adverse events such as longer hospitalizations, increased antimicrobial resistance, morbidity and mortality, economic and psychological consequences [1]. In developed countries, HAIs affect 5–15% of hospitalized patients and can reach 9–37% of those admitted to intensive care units [2]. The situation is more dire in low-income countries where health care systems are often less developed [3, 4]. HAIs prevention must be a priority as decreasing their incidence [1, 3].

The hands of Healthcare Workers (HCWs) are the most common vehicle for the transmission of microorganisms from patient to patient within the healthcare environment. For instance, *Klebsiella Sp*, Methicillin Resistant *Staphylococcus aureus* (MRSA), *Clostridium difficile* and Gram-negative bacilli are some of the organisms that are likely to be found on HCWs hands [2]. Hand hygiene (HH; i.e. washing hands with soap and water, or disinfection using alcohol based hand rub solution: ABHR) is essential to prevent and control HAIs as it reduces microbial colonization and direct transmission from patient to patient [5, 6]. However, compliance with HH among HCWs is frequently reported as poor and is usually estimated as less than 50% [7]. Average compliance with HH recommendations varies between hospital wards, among professional categories, working conditions as well as according to the definitions used in different studies [8–10].

To promote HH, World Health Organization (WHO) has published “My five moments for hand hygiene”, describing the HH opportunities, which are the moments during healthcare activities when HH is necessary to interrupt germ transmission by hands: Moment 1- before touching a patient, Moment 2- before clean/ aseptic procedure, Moment 3- after body fluid exposure risk, Moment 4- after touching a patient and Moment 5- after touching patient surroundings [8, 11]. The compliance to HH is the proportion of times that HCWs perform HH of all five observed opportunities [12]. The WHO and the Centers for Disease Control and Prevention (CDC) recommend that HCWs wash their hands with soap and water, when visible soiling is present for 40 to 60 s. When hands are not visibly soiled, alcohol based hand rub (ABHR) for 20 to 30 s is recommended [13].

Despite the evidence of benefits of good HH practices and the relative cost-effectiveness and simplicity of this procedure, compliance remains a challenge, and more so in developing countries [2, 14]. Different factors have been described to influence HH compliance. For instance, the lack of appropriate infrastructure, the cultural backgrounds and even religious beliefs can play an important role in hindering good practices [15]. The professional category such as doctors, nurses and situations requiring wearing of gowns and gloves can also determine poor HH compliance [2].

Even tool in obstetric and gastrointestinal surgery exist in Benin, this study is to the best of our knowledge, the first to document the utilization of the WHO HH observation method [16]. Therefore, the aim of the study was to assess the rate of HH compliance among HCWs using the WHO My five moments for hand hygiene tool in obstetric and gastrointestinal surgery.

## Methods

### Design and setting

Direct observation of HCWs during patient care activity by trained and validated observers is recognized as the gold standard for hand hygiene monitoring [12]. We conducted this prospective observational study for 2 months (June–July 2018), at six hospitals in the south of Benin to allow enough time for the collection of 200 opportunities according to the sample size recommended by the WHO.

These hospitals belonged to intermediate (i.e. first reference in the department; Centre hospitalier Universitaire Départemental de l’Ouémé Plateau) and central (i.e. tertiary care; Centre Hospitalier Universitaire Hubert Koutoukou de Maga CNHU, Centre Hospitalier Universitaire de la Mère et de l’Enfant CHUMEL, Bethesda, Centre Hospitalier de zone Suru Lere and Centre Hospitalier Universitaire de Zone d’Abomey-Calavi) level of health care in Benin. Observations were conducted in two selected settings: obstetric (delivery room and ward) and gastrointestinal surgery (operating room and ward).

### Participants

All HCWs (physicians, nurses-auxiliaries, and other HCWs) present in the room were eligible for observation if they provided care to patients. Observations were conducted unobtrusively during routine patient care, mostly during the day but also during the night. HCWs were unaware of being observed to minimize “Hawthorne effect”. Additionally, observers did not provide details of the study procedures for HCWs.

### Outcomes

We used the previously validated WHO “My five moments for hand hygiene” observation tool to collect HH opportunities and calculate the primary outcome: HH compliance. The term “opportunity” for HH is defined as a “moment during healthcare activities when HH is necessary to interrupt germ transmission by hands” [14].

Each application of alcohol hand rub or hand washing with soap and water was regarded as a complied HH opportunity. For instance, any HCW who decontaminated their hands immediately after a contact with a patient and then directly attended another patient without touching any object (for example no touching of door handles or any other patients) was considered to have complied with HH practices in relation to the second patient [8, 14].

Secondary outcomes included determinants of compliance, the observation of HCWs adherence to basic HH parameters such as short clean nails and white tips < 3 mm long, use of nail polish (bare nails), absence of jewellery (rings, bracelets and watches) and the duration of HH.

### Variables and data collection

To gather the required information, we developed a 3-part paper observation grid including: (A) adherence to basic HH parameters, (B) “My 5 moments for HH” tool and (C) techniques and duration of HH. Pre-test observation was done out of the study period for 2 days with 2 voluntary nurses in each hospital to calibrate this observation grid.

The observer team was composed of eleven medical students. The author and a specialist of hygiene and infection control, working in one of the six hospitals, previously trained the students. Each observer was allocated to one hospital and one setting (obstetric section or gastrointestinal surgery). Before the beginning of the data collection, observers underwent 1) a 3-day coaching on HH and the use of the two grids, and 2) a 1-week training in each hospital to familiarize themselves with the infrastructure, the procedures, local HH policy (use of clean water, soap and alcohol) and the personnel.

During the training, we used the WHO training film which describe possible situations during observations sessions. Each observer completed the observational form separately while observing the same HCW and the same care sequence. Results were then compared and discordant notifications were discussed. This process was repeated until concordance is reached in terms of the number of hand hygiene opportunities and hand hygiene actions that occurred. During the study period, observer team received periodic reinforcement of education (twice per month) focusing on technical issues to conduct observations according to Sax H. method [12].

Trained observers completed one observation grid per observation session. The observation session is a period of 20 ± 10 min in a care setting [14]. HCWs could be observed during several sessions. Each HCW was observed for a maximum of four HH opportunities during the observed care session according to WHO recommendations [1].

In addition, we used monthly ward infrastructure survey to collect data about infrastructure at ward level. This grid is divided into 28 indicators with questions about resources and facilities for HH practices such as: sink, water and alcohol, presence or absence of reminders in workplace. At the end of each month, a grid was completed by observers and senior nurses of each hospital. **Statistics.**

The data were initially recorded on paper and then entered directly into Epidata 3.1. Analysis was done using SPSS version 22 software. Analyses of all variables were conducted overall, and the results presented by frequencies and percentages. Compliance was calculated by dividing the numbers of times hand hygiene was performed by the total number of opportunities.

The chi square test was used to test associations between variables if the expected frequency was above 5. If the expected frequency below 5, Fisher’s exact test was used. In all analyses, *p* value ≤0.05 was considered to be statistically significant.

## Results

### General characteristics

For 60 days, we observed 432 HCWs around six hospitals (57.9% women versus 42.1% males). Most of them were nurses (55.6%) and auxiliaries (25.7%), followed by physicians (13.4%) and others 5.3% (i.e. technicians, anesthetists and student physicians or nurses). Table 1 shows the characteristics of observed HCWs.

**Table 1:** Characteristics of observed HCWs

Characteristics	Hosp A	Hosp B	Hosp C	Hosp D	Hosp E	Hosp F	Total
**Professional category**							
Physicians n (%)	26 (17.5)	1 (2.9)	6 (9.8)	15 (17.6)	4 (7.4)	6 (12.2)	58 (13.4)
Nurses and auxiliaries n (%)	117 (78.5)	33 (97.1)	42 (68.9)	67 (78.8)	50 (92.6)	42 (85.7)	351 (81.3)
Others n (%)	6 (4.0)	0 (0.0)	13 (21.3)	3 (3.6)	0 (0.0)	1 (2.0)	23 (5.3)
**Total** n (%)	149 (100.0)	34 (100.0)	61 (100.0)	85 (100.0)	54 (100.0)	49 (100.0)	432 (100.0)

Hosp F=Intermediate level, and the others = central level. A:CNHU-HKM, B:BETHESDA C: CHU-SURU-LERE, D: CHUZ ABOMEY-CALAVI, E: CHUMEL, F: CHUZ PORTO-NOVO.

### Hand hygiene compliance and its determinants

One thousand three hundred fifteen HH opportunities were observed. The overall HH compliance was 33.3% [30.8-CI-35.8], with no significant difference between professional categories. Indeed, compliance was 32.4% among physicians, 34.2% among nurses and 25.6% for other HCWs (*p* = 0.705). The compliance was higher among females than males. (40.5% vs. 26.4% *p* < 0.001).

In maternity, HH compliance reached 49.4% [44.9-CI-53.9] while surgery services scored 24.3% [21.4-CI-27.2] (*p* < 0.001). HH compliance before patient contact was 25.2, 33.0% before a clean or aseptic procedure, 37.5% after patient contact 29.1% after contact with patient surroundings and 54.5% after body fluid exposure. The compliance rate did not significantly differ according to the shift (39.4% in the afternoon, 32.4% in the morning, and 50.0% in the night *p* = 0.113) (Table 2).

**Table 2:** Hand hygiene compliance among HCWs in Benin

Characteristics	Hand Hygiene opportunities (n)	Hand Hygiene actions (n^a^)	% Compliance	*P* value
**Overall Wards**	1315	438	33.3	
Surgery visceral	841	204	24.3	*P* < 0.001
Maternity	474	234	49.4	
**Professional category**				
Physician	139	45	32.4	*P* = .705
Nurses	752	257	34.2	
Auxiliary nurse	385	126	32.7	
Others	39	10	25.6	
**Sex**				
Male	671	177	26.4	*P* < 0.001
Female	644	261	40.5	
**Shift**				
Morning	1161	376	32.4	*P* = .113
Afternoon	142	56	39.4	
Night	12	6	50.0	
**WHO five moments**				
Moment 1	393	99	25.2	*P* < 0.001
Moment 2	209	69	33.0	
Moment 3	77	42	54.5	
Moment 4	509	191	37.5	
Moment 5	127	37	29.1	
**Care room**				
Bandage area	571	267	46.8	*P* < 0.001
Hospitalization	691	139	20.1	
Others	53	32	60.4	

Five moments: 1-before touching patient, 2-Before clean/aseptic procedure, 3-after body fluid exposure risk, 4-after touching a patient, 5-after touching patient surroundings.

### Hand hygiene technique and duration

**Fig. 1:**
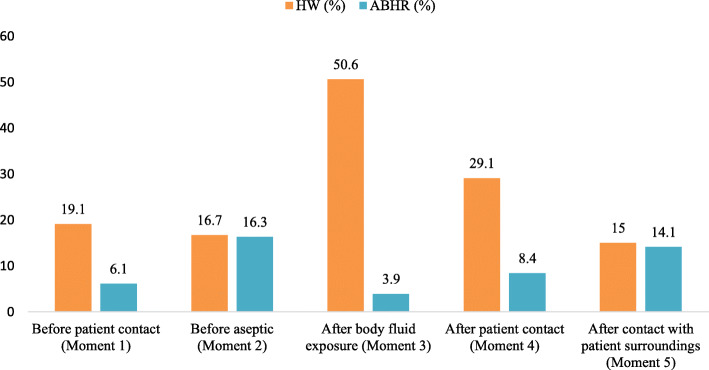
Hand hygiene actions by indications among HCWs (Benin 2018)

When HH was applied, technique and duration were often not appropriate. Hand washing (HW) remains the most preferred action. Among 438 HH actions, HW was performed in 316 cases (72.1%) while ABHR was performed in 122 cases (27.9%). 0% used both technics ABHR and soap and water. After body fluid exposure when hands were visibly soiled, hand washing was performed in 50.6% of cases while ABHR was for 3.9%. Before aseptic action and for other moments hand washing was always the first action of compliant HCWs through six hospitals (Fig. 1).

Hand hygiene duration was almost never fully observed by HCWs.(< 6.0% for ABHR and 0.5% for hand washing). The median duration for hand washing was 10 ± 7 s (instead of 40 to 60 s) and 9 ± 6 s (instead of 20 to 30 s) for ABHR (Table 3).

**Table 3:** Hand hygiene duration among compliant HCWs in June–July 2018

Hand Hygiene Duration	Frequency (n)	Percentage (%)
**Wash duration in seconds (s)**	Median washing (10 ± 7)	
Less than 40	199	99.5
More than 40	1	0.5
**Alcohol rub duration (s)**	Median ABHR (9 ± 6)	
Less than 20	78	94.0
More than 20	5	6.0

### Adherence to basic hygiene parameters

The adherence to basic hygiene parameters regarding nails and jewellery among HCWs was higher than the HH compliance.

In total, more than 77% (*n* = 334) had their hands free of jewellery and 91% (*n* = 393) of them had natural short and without polish fingernails. Only 1.8% (*n* = 8) of healthcare professionals had nails extensions**.**

### Hand hygiene infrastructures

Clean water and alcohol based hand rub was only available half of time in all of six hospitals, sinks were often inconveniently located: out of the patient room or the point of care. None of the hospitals provided single-use towels. Only two hospitals displayed WHO posters explaining correct use of hand rub and handwashing technique. Observed hospitals did not organize training sessions about HH for HCWs. (Table 4) summarizes HH infrastructures for six hospitals.

**Table 4:** Hand hygiene infrastructures in six hospitals, 2018

Characteristics	Hosp A	Hosp B	Hosp C	Hosp D	Hosp E	Hosp F
Is Running water available	A	I	I	I	I	I
Is water visibly clean	Yes	Yes	Yes	Yes	Yes	Yes
Kind of taps available	Hand- Operated	Hand-operated	Hand-operated	Hand-operated	Hand-operated	Hand-operated
Are disposable towels available at all sinks	No	No	No	No	No	No
Is soap available at all sinks	I	Yes	Yes	Yes	Yes	I
Is an ABHR available (%)	100	50	50	100	50	50
Are posters illustrating HH technique beside each sink	No	No	Yes	No	Yes	No
Is any other type of reminder on HH available on this ward?	No	No	No	No	No	No
Are audits on HH compliance periodically performed?	No	No	No	No	No	No

*I* Intermittently = Unavailability half of time. *ABHR* Alcohol based Hand Rub, *A* Always

## Discussion

Hand hygiene is one of the foremost techniques to reduce HAIs [17]. However, overall HH compliance was 33.3% in this observational study. The behavior of HCWs showed a tendency towards self-protection, that is, with a higher compliance rate after body fluid exposure risk and after patient contact than before touching patient. The adherence to HH parameters regarding hair, nails and the use of jewellery was better than over all compliance. HH infrastructures were deficient or inconstantly present at the point of care. This multicenter observational study is the first in Benin which assessed HH compliance according to WHO recommendations. However, this also makes it difficult to compare our results with prior findings.

Overall HH compliance was lower (33.3%) than findings of authors from Nigeria (65.3%), Brazil (46.7%) and Saudi Arabia (50.3%) [18]. Hand hygiene compliance seems better in maternity care room. The possible reason might due to the possible regular availability of water and washing agents in maternity compared to the gastrointestinal surgery. Luangasanatip et al. found in a systematic review and network meta-analysis of 41 hand hygiene studies that baseline compliance with HH among HCWs was on average only 38.7% [19] . A 24 h observational study in Ethiopia showed lower (22.0%) compliance rates than in our study but they collected less opportunities than in our study [4]. In Benin, the WHO’s multimodal hand hygiene improvement strategy is not implemented yet [20]. Many studies suggest that HH intervention such as the multimodal strategy of WHO has a great potential to improve HCW compliance [18, 21].

The compliance was low across all professional categories. However, nurses tend to be more compliant than auxiliary nurses and physicians (*P* = 0.705). This observation is concordant with the findings of other studies [22, 23]. In the literature, compliance with HH among nurses are better than doctors [24, 25]. In a multicenter study done in 5 countries (Costa Rica, Italy, Mali, Pakistan and Saudi Arabia), physicians had the lowest and the nurses had the highest compliance except for Mali [8]. The lower compliance among physicians could be explained by the absence of a team leader acting as a role model for other colleagues [26]. Medical students reported feeling strongly influenced by negative role models thereby abstaining from compliance with HH guidelines [27]. Erasmus et al. showed that physicians seem to be skeptical or less convinced than other professional categories about the effectiveness of HH to reduce HAI or to limit the spread of antimicrobial resistance and improve patient safety [27, 28]. Some studies even suggested turning nurses into ambassadors of hand hygiene programs [29, 30]. In most of the cases, nurses are aware of the rationale for hand hygiene procedures and represent a large working group that performs the greatest amount of direct patient care. If nurse behavior can change, the impact on the healthcare system will be significant [31]. Clearly, there is a need to develop strategies to improve physician’s compliance [17]. In our setting for instance the best way will to implement in physicians 7 years cursus, hand hygiene courses. No study has been undertaken in Benin about the importance of including HH program in the curriculum. But it has been done elsewhere. For instance Chakravarthy et al. found that “the knowledge levels of medical students about HH is far below expectations; the administrators should take upon themselves to include this topic in the educational curriculum” [32].

The compliance was low before patient contact (25.2%) and after contact with patient surroundings (29.1%). Dancer et al. argue that environmental cleaning needs to be improved generally and specifically at hand touch sites [33]. Hand touch sites with the highest risk to patients are those which are next to the patient, for example, bedrails, lockers, over beds tables and door handles [33, 34]. Carling et al. found that more than half of the inanimate objects such as those previously listed were not microbiologically clean when screened [35]. By contrast, HCWs were more compliant after body fluid exposure and after patient contact (*p* < 0.001). This finding is in line with an observational study in Kuwaiti hospitals that suggested that HCWs perform HH for their own protection, rather than to protect their patients [36]. Self-protection tendency has been identified in multiple studies [36, 37].

Despite recommendation of the Centers for Disease Control (CDC) and WHO guidelines on HH in healthcare to use ABHR solution as the preferred means for routine hand hygiene [21, 38], its use remains poor. Among 438 HH actions, only 122 (27.9%) used alcohol based hand rub solution while 316 (72.1%) performed hand washing. This finding could be explained by lack of knowledge concerning its benefits, and also lack of infrastructure in our hospitals. Moreover, the few HCWs who washed their hands, did so inefficiently. The median time for hand rubbing was 9 s and only 6% respected the correct time. There was no gain in reducing bacterial counts from hand rubbing longer than 30 but a 30 s application is usually sufficient [39]. By contrast, the median time for hand washing was 10 s and 0.5% respected the correct duration. Reported reasons for not respecting HH duration and technique include too many opportunities per hour, or simply ignorance of guidelines or forgetfulness [7, 29, 40].

Unfortunately 22.7% of the HCWs still use long natural, polished or artificial fingernails when caring patients. This is strongly discouraged by the WHO because the majority of flora on the hands are found under and around the fingernails [13]. Artificial and long polished nail harbor harmful bacteria and studies described difficulty in their elimination with cleaning. Alcohol-based hand rub solution cleared pathogens from both artificial and natural nails better than antimicrobial soap [41, 42]. However, jewellery must be kept to a minimum. Jeweled rings or rings with stones should not be worn, a plain wedding ring is permitted [43].

There are serious barriers to compliance in the observed hospitals. For instance, the compliance rate was particularly low (20% vs 46,8%; *p* < 0.01) in hospitalization rooms compared to the bandage room where we can intermittently find sinks and other infrastructures. By contrast, in all of 6 hospitals, supplies were not placed at key location throughout patient room. Absence or irregularity of HH supplies can sometimes discourage personal to wash their hands when indicated [23]. Limited access to hand hygiene supplies such as soap or alcohol hand rub solutions are challenges compliance [23]. Cantrell et al. found a positive correlation between compliance rates and the presence of a sink in the patient’s room [44]. In addition, there were no poster and other visual reminders for HH at work in some hospitals which may help increase compliance. The present audit is the first one in all of these hospitals, making it very important for future hospital planning, management and patient safety. Successful promotion of hand hygiene strategies (e.g., making hand hygiene products available at the point of care, allocate special budget for HH, improvement of HH education among HCWs) should be considered in all of these hospitals.

### Strengths and limitations

The main strength of this study is the settings: it is the first study to document the utilization of the WHO HH observational method. Moreover, it included two wards in six different hospitals. Despite the limited numbers of observations during the night shift and weekends and the fact that data collection did not record the use of gloves, this study provides HCWs with reliable information about HH practice in Benin. The present study was conducted using direct observation method, the most reliable method for measuring the rate of adherence to HH [45]. Observers were trained and used validated data collection forms. A potential bias associated with observation is called the “Hawthorne effect”. HCWs have opportunities to change their behavior when they know that they are being observed [46]. So, they were not aware they were being observed to diminish this effect.

This study was the first step of MUltidisciplinary STrategy for Prevention and Infection Control (MUSTPIC) project. This project aims to increase HCWs compliance by implementation of WHO Multimodal Strategy which includes 5 keys components: system changes, HCWs training and education, evaluation and feedback, reminders in the workplace, and promotion of an institutional safety climate. Next steps include a qualitative assessment of factors underlying HH compliance with interviews among HCWs.

## Conclusion

This study showed a poor HH compliance among different HCWs in surgery in Benin. As HH is an influential and cost-effective way of reducing HAIs, it should become an educational priority in Benin health care settings with the promotion of the WHO multimodal hand hygiene strategy: “Hand care is safer care”. Access to HH resources should be emphasized as an integral part of HH improvement strategy.

## Data Availability

The data supporting the results of this study are included within the article and its additional files.

## Abbreviations

HCWs: Health care workers
WHO: World health organization
HAIs: Healthcare associated infections
HH: Hand hygiene
HHO: Hand hygiene opportunities
ABHR: Alcohol based hand rub
CI: Confidence interval
HW: Hand washing
